# Acanthosis nigricans and acrochordons as clinical markers of metabolic disturbance during pregnancy: a prospective cohort study

**DOI:** 10.3389/fmed.2025.1607429

**Published:** 2025-09-12

**Authors:** Erika Ilatov, Tamar Eshkoli, Aida Mashal, Lior Yahav, Ariel Binyaminov, Noga Borochov, Adi Y. Weintraub, Amir Horev

**Affiliations:** ^1^Department of Obstetrics and Gynecology, Soroka University Medical Center, Beersheba, Israel; ^2^Faculty of Health Science, Ben-Gurion University of the Negev, Beersheba, Israel; ^3^Pediatric Dermatology Service, Soroka University Medical Center, Beersheba, Israel

**Keywords:** acanthosis nigricans, acrochordon, pregnancy outcome, metabolic disturbance, dermatological manifestations

## Abstract

**Background:**

This study investigated the prevalence of acanthosis nigricans and acrochordons as potential markers of diabetes or insulin resistance in diabetic and non-diabetic pregnant women, and examined their potential association with pregnancy complications.

**Methods:**

This prospective cohort study was conducted at a tertiary university medical center and included 62 pregnant women with diabetes during pregnancy and 58 non-diabetic controls. Maternal demographics, obstetrical history, and neonatal outcomes were collected. The presence of acanthosis nigricans and acrochordons was assessed at multiple body sites. Multivariable logistic regression analysis was performed to identify obstetrical complications and perinatal outcomes that are independently associated with acrochordons.

**Results:**

Acanthosis nigricans was more prevalent among women with diabetes, particularly in the axilla (59.7% vs. 32.8%, *p* = 0.004), under the breast (46.8% vs. 19%, *p* = 0.002) and in the lower abdomen (35.5% vs. 25.5%, *p* = 0.021). Similarly, acrochordons were significantly more common in diabetic patients, with a higher prevalence at various sites (*p* < 0.001). In multivariable regression analysis, diabetes during pregnancy and maternal age were independently associated with a high prevalence of acrochordons.

**Conclusion:**

Pregnant women with gestational diabetes showed a significantly higher prevalence of acanthosis nigricans and acrochordons compared to those without. This association suggests that acanthosis nigricans and acrochordons may be potential indicators of underlying insulin resistance during pregnancy.

## 1 Introduction

Acrochordons (skin tags), are benign, small, soft, and often pedunculated skin tumors frequently found on the neck, axilla, and groin. They represent protrusions of loose fibrous tissue and typically range in size from 2 mm to 6 mm in diameter. While they are usually skin-colored, larger and hyperpigmented lesions may also be observed during dermatological examinations. Although acrochordons are benign and of little clinical significance, studies have reported an increased prevalence in individuals with systemic conditions such as acromegaly and colonic polyps (1, 2). In 1951, Touraine proposed a possible association between acrochordons and diabetes mellitus (DM) (3). Since then, several clinical studies have been conducted to investigate this hypothesis, yielding conflicting results (4–7). Additionally, acrochordons have been observed with increased frequency during the second trimester of pregnancy, often regressing postpartum (8).

Acanthosis nigricans (AN) is a well-recognized cutaneous marker of insulin resistance (9). It has been linked to severe insulin resistance, including in obese women with gestational diabetes mellitus (GDM) (9). Daitchman et al. (10) found that women with AN had higher body weight and were nearly twice as likely to require insulin therapy, with a higher daily dose.

Diabetes during pregnancy is classified into two main categories: pregestational diabetes mellitus (including type 1 and type 2 diabetes) and GDM. GDM is further divided into GDMA1, which is well-controlled without medication, and GDMA2, which requires pharmacological treatment to achieve euglycemia (11). Women with GDM are at increased risk of pregnancy complications such as preeclampsia and cesarean delivery. Moreover, their offspring are at a higher risk of macrosomia, neonatal hypoglycemia, and birth trauma (11). Additional factors associated with diabetes during pregnancy or impaired glucose metabolism postpartum include fasting plasma glucose (FPG), 1-h plasma glucose, hemoglobin A1C (HbA1c) at GDM diagnosis, maternal age, pregestational weight and maximum weight, pregestational body mass index, maternal birth weight, family history of diabetes in first-degree relatives, and prenatal weight (12).

A previous study demonstrated that pregnant women presenting with both acrochordons and acanthosis nigricans had a 4.8-fold higher likelihood (95% CI: 1.9–11.7) of developing GDM compared to those without these dermatological findings (13). However, there is a lack of data regarding the potential benefits of early screening and diagnosis of diabetes among pregnant women with acrochordons and AN to prevent pregnancy-related complications.

In the present study, we aim to evaluate the potential relationship between acrochordons and AN with diabetes during pregnancy, the need for insulin treatment, pregnancy outcomes, and the risk of impaired glucose metabolism in the postpartum period.

## 2 Materials and methods

### 2.1 Study design

A prospective cohort study was conducted at Soroka University Medical Center (SUMC) to evaluate the potential association between acrochordons and GDM. SUMC is a tertiary medical center and the sole facility providing obstetric admission and delivery services for the entire Negev sub-district, where approximately 98% of all deliveries take place, with an annual average of around 17,000 births. The study was conducted in accordance with the declaration of Helsinki and all appropriate amendments. The study was reviewed and approved by the local Ethics Committee of SUMC, Israel (Approval Number 0016-22-SOR). Written informed consent was obtained from each patient before entering the study.

### 2.2 Study population

The study population included pregnant women diagnosed with diabetes during pregnancy, who were recruited from the day-hospitalization clinic and the obstetric emergency room (ER). The control group consisted of pregnant women, without diabetes, randomly recruited from the obstetric and gynecological ER, high-risk pregnancy departments, and the day-hospitalization clinic. All participants provided informed consent before undergoing an examination for the presence of acrochordons and acanthosis nigricans (AN). Data regarding diabetes status and pregnancy parameters were collected and recorded.

To assess the number of acrochordons, a physician conducted a physical examination at four anatomical locations: the neck, inframammary region, axillae, and groin. The total number of acrochordons s was documented, along with the presence of AN. The diagnosis of acrochordons and acanthosis nigricans was performed by trained physician under the direct supervision and instruction of a board-certified dermatologist, including standardized criteria for identification and grading. In relevant cases, such as those first examined during the first trimester, follow-up assessments were conducted. At the time of delivery, pregnancy outcomes were retrieved from the computerized medical records at SUMC. Once all participants had given birth, the collected data were analyzed.

### 2.3 Statistical analysis

The initial analysis included descriptive statistics, such as means, standard deviations (SD), and graphical representations. Advanced statistical analyses were performed using parametric tests where applicable. Continuous variables with a normal distribution were presented as mean ± SD and compared between study groups using the independent *t*-test. Non-normally distributed continuous variables were expressed as medians with interquartile ranges (IQR) and analyzed using the Mann–Whitney test. Categorical variables were presented as counts and percentages and analyzed using the chi-square test or Fisher’s exact test, as appropriate. A two-sided *p*-value of <0.05 was considered statistically significant.

## 3 Results

### 3.1 Diabetic vs. non-diabetic

During the study period, 119 women met the inclusion criteria, of whom 62 were diagnosed with diabetes during pregnancy, while 58 served as non-diabetic controls. Women with diabetes during pregnancy were significantly older than those in the control group, with a mean age of 32.9 ± 5.48 years compared to 28.3 ± 5.86 years, respectively (*p* < 0.001) (Table 1). Additionally, the prevalence of obesity was significantly higher among diabetic women, observed in 74.1% of cases compared to 49.1% in the control group (*p* = 0.01). Hypothyroidism was significantly more common in the diabetic group, affecting 11.1% of women, whereas no cases were reported in the control group (*p* = 0.014). A history of GDM in previous pregnancies was significantly more frequent in the diabetic group, with 16.1% of women reporting a prior diagnosis compared to only 3.4% in the control group (*p* = 0.03) (Table 1). Dermatologic findings were notably more prevalent among women with diabetes. The prevalence of acanthosis nigricans was significantly higher in the diabetic group, particularly in specific body areas. The axilla was the most affected site, with 59.7% of diabetic women exhibiting acanthosis nigricans compared to 32.8% in the control group (*p* = 0.004) (Table 2). The condition was also more common under the breast, where it was observed in 46.8% of diabetic women compared to 19% of controls (*p* = 0.002), and in the lower abdomen, where it was present in 35.5% of diabetic women versus 25.5% in the control group (*p* = 0.021). Similarly, acrochordons were significantly more prevalent among diabetic women, with a broader distribution across multiple body sites, a difference that reached statistical significance (*p* < 0.001) (Table 3).

**Table 1:** Clinical and demographical characteristics of the study population.

Clinical and demographical characteristics	Diabetes (*n* = 62)	Control (*n* = 58)	*p*-value
Gestational age at recruitment	32.41 ± 7.49	35.56 ± 4.06	0.005
Gravidity	3 (8)	2 (11)	0.32
Parity	1 (8)	1 (6)	0.89
Maternal age	32.9 ± 5.48	28.34 ± 5.86	<0.001
Weight at recruitment	85.01 ± 16.73	79.22 ± 17.02	0.066
Weight before pregnancy	73.91 ± 16.34	68.53 ± 18.85	0.27
Height	1.6 ± 0.07	1.62 ± 0.05	0.18
BMI	32.9 ± 6	29.85 ± 6.46	0.01
Obesity	40 (74.1%)	28 (49.1%)	0.01
Jewish ethnicity	42 (66.7%)	32 (55.2%)	0.26
Hypothyroidism	7 (11.1%)	0	0.014
Asthma	4 (6.3%)	2 (3.4%)	0.68
History of GDM	10 (16.1%)	2 (3.4%)	0.03
Abortions	16 (25.8%)	10 (17.2%)	0.27

**Table 2:** Distribution of acanthosis nigricans across different body sites in the study population.

Acanthosis nigricans	Diabetes (*n* = 62)	Control (*n* = 58)	*p*-value
Neck	19 (30.6%)	9 (15.5%)	0.056
Axilla	37 (59.7%)	19 (32.8%)	0.004
Under the breast	29 (46.8%)	11 (19%)	0.002
Under the abdomen	22 (35.5%)	9 (25.5%)	0.021
Groin	35 (56.5%)	27 (46.6%)	0.36
Sum	2 (5)	1 (5)	0.002
75 percentiles	29 (46.8%)	11 (19%)	0.002

**Table 3:** Distribution of acrochordons across different body sites in the study population.

Acrochordons	Diabetes (*n* = 62)	Control (*n* = 58)	*p*-value
Neck	4 (15)	1 (14)	<0.001
Axilla	1 (10)	0 (4)	<0.001
Under the breast	1 (27)	0 (4)	<0.001
Under the abdomen	0 (6)	0 (4)	0.01
Groin	0 (14)	0 (5)	0.04
Sum	8 (54)	2 (22)	<0.002
75 percentiles	28 (45.2%)	7 (12.1%)	<0.001

### 3.2 Pregnancy complications and neonatal outcomes

Pregnancy complications and neonatal outcomes among patients with diabetes during pregnancy and the control group are presented in Table 2. Labor induction was performed more frequently among diabetic women, with a rate of 39.7% compared to 22.4% in the control group (*p* = 0.05). Despite the maternal differences, neonatal outcomes showed no significant variations between the two groups. APGAR scores at both 1 min and 5 min were comparable, and neonatal birth weight did not differ significantly, with a mean weight of 3,123 ± 634 g in the diabetic group compared to 3,040 ± 786 g in controls (*p* = 0.52) (Table 4).

**Table 4:** Pregnancy complications and neonatal outcomes among patients with diabetes during pregnancy and the control group.

Pregnancy complications and neonatal outcomes	Diabetes (*n* = 62)	Control (*n* = 58)	*p*-value
Placental complications	5 (8.1%)	5 (8.6%)	0.99
Hypertensive disease	7 (11.1%)	7 (12.1%)	0.89
IUGR^a^	5 (7.9%)	6 (10.3%)	0.75
Induction of labor	25 (39.7%)	13 (22.4%)	0.05
Preterm delivery	6 (9.7%)	7 (12.1%)	0.77
IUFD	1 (1.6%)	0	0.99
Polyhydramnios	1 (1.6%)	2 (3.4%)	0.6
PPROM/PROM	4 (6.3%)	9 (15.5%)	0.14
Gestational age	37.3 ± 3.66	38.38 ± 2.31	0.06
Cesarean section	26 (41.9%)	18 (31%)	0.25
Neonatal weight	3123.27 ± 634.2	3040.16 ± 786.42	0.52
APGAR 1 min	9 (8)	9 (8)	1
APGAR 5 min	10 (9)	10 (9)	1
PH	7.28 ± 0.1	7.29 ± 0.09	0.51
Episiotomy	3 (4.8%)	1 (1.7%)	0.61
Perineal tear	10 (16.1%)	14 (24.1%)	0.36
Epidural	30 (48.4%)	36 (61.4%)	0.19
Neonatal male sex	35 (57.4%)	28 (49.1%)	0.46
LGA	5 (8.1%)	7 (12.1%)	0.55

a IUGR, intrauterine growth retardation; IUFD, intra uterine fetal demise; PROM, premature rupture of membranes; LGA, large for gestational age.

### 3.3 Multivariable logistic regression analysis

Multivariable regression analysis identified both diabetes during pregnancy and advanced maternal age as independent predictors of a higher prevalence of acrochordons (Table 5). Diabetes in pregnancy remained a significant risk factor even after adjusting for age and other confounders, with an adjusted odds ratio of 5 (95% confidence interval: 1.68–14.88, *p* = 0.004).

**Table 5:** Results of multivariable logistic regression analysis with 75 percentiles of acrochordons as the outcome.

Variables	Adjusted OR	95% CI		*p*-value
		Lower	Upper	
Diabetes	5.005	1.683	14.885	0.004
Gestational age at recruitment	0.988	0.901	1.083	0.790
Obesity	1.375	0.458	4.127	0.570
Age	1.140	1.035	1.257	0.008
Hypothyroidism	0.532	0.083	3.405	0.506
Previous GDM	0.942	0.223	3.982	0.936

## 4 Discussion

The results of this study confirm that pregnant women with diabetes exhibit a higher prevalence of both acrochordons and acanthosis nigricans, particularly in areas such as the axilla, inframammary region, and lower abdomen. Multivariable regression analysis further identified diabetes during pregnancy and advanced maternal age as independent factors associated with an increased frequency of acrochordons. These findings suggest that acrochordons and acanthosis nigricans may serve as early, visible indicators of metabolic disturbances in pregnant women, facilitating the early detection and monitoring of insulin resistance and related complications.

Our findings align with previous research that demonstrated an association between acanthosis nigricans and insulin resistance, particularly in individuals with obesity and GDM (10). Similarly, prior studies have suggested that acrochordons may be linked to metabolic disturbances, although data on pregnant populations remain limited (3–7). The increased prevalence of these dermatologic conditions among women with diabetes during pregnancy supports their role as clinical markers of insulin resistance. Moreover, previous research has indicated that the presence of both acrochordons and acanthosis nigricans in pregnant women is associated with a significantly higher risk of developing GDM (13), reinforcing the potential clinical relevance of these findings. The current study adds to the growing body of evidence on the topic.

While our findings support the association between dermatologic markers and insulin resistance, we did not find notable differences in neonatal birth weight or APGAR scores between diabetic and non-diabetic groups. Previous studies have explored the relationship between insulin resistance severity and dermatologic markers, such as acanthosis nigricans (10), our study did not directly quantify insulin resistance levels. Further research is needed to determine whether these dermatologic markers correlate with neonatal outcomes or serve only as characteristics of metabolic disturbance.

The identification of acrochordons and acanthosis nigricans as potential markers of metabolic disturbances during pregnancy provides clinicians with a valuable, non-invasive screening tool. Integrating dermatologic assessments into routine prenatal care may enhance early detection and intervention strategies for women at risk of diabetes-related complications. Early identification of metabolic disturbances can contribute to improved pregnancy outcomes by allowing for timely interventions such as dietary modifications, glucose monitoring, and pharmacological management (11, 14). Previous studies have emphasized that proactive management of insulin resistance during pregnancy may reduce the risk of adverse maternal and neonatal outcomes (11, 14), reinforcing the importance of dermatologic markers as potential early warning signs.

The strengths of this study include its prospective cohort design and the use of standardized clinical assessments for dermatological findings. Additionally, the study was conducted at a tertiary medical center, ensuring a well--diverse patient population. However, certain limitations should be acknowledged. The study was conducted at a single medical center, which may limit the generalizability of the findings. While confounders such as maternal age and obesity were accounted for, other potential influences, including genetic predisposition and additional metabolic disorders, were not comprehensively assessed. Additionally, Data on polycystic ovarian syndrome (PCOS) were incompletely documented. As it is a diagnosis of exclusion and several women had menstrual irregularities together with hyperandrogenism without a formal diagnosis, its prevalence in our study population was likely substantially underestimated, precluding reliable subgroup analysis. Further multicenter studies with larger sample sizes are needed to validate these findings and elucidate underlying mechanisms.

## 5 Conclusion

Pregnant women with gestational diabetes showed a significantly higher prevalence of acanthosis nigricans and acrochordons compared to those without. This association suggests that acanthosis nigricans and acrochordons may be potential indicators of underlying insulin resistance during pregnancy. Early recognition of these skin changes may provide opportunities for preventing or mitigating pregnancy complications associated with gestational diabetes. Ongoing research should further explore the predictive value of these dermatologic markers and their role in refining screening and management protocols for gestational diabetes and related metabolic disorders.

## Data Availability

The raw data supporting the conclusions of this article will be made available by the authors, without undue reservation.
